# Culturing surplus poor-quality embryos to blastocyst stage have positive predictive value of clinical pregnancy rate 

**Published:** 2014-09

**Authors:** Hai Bo Zhu, Zhi Hong Zhang, Elfateh Fadlalla, Rui Xue Wang, Dong Feng Geng, Rui Zhi Liu

**Affiliations:** *Reproductive Medical Center, First Hospital of Jilin University, Changchun, Jilin, China. *

**Keywords:** *Blastocyst*, *Embryonic development*, *Pregnancy rate*, *Fertilization in vitro*, *Sperm injections*, *Intracytoplasmic*

## Abstract

**Background:** Clinical reproductive centers produce large amounts of surplus poor-quality embryos annually, how to maximize the use of these embryos, and which of them have the potential to develop into blastocyst stage and influencing factors were lack of systematic research.

**Objective:** To investigate the fate of surplus poor-quality embryos which were cultured to obtain blastocyst, determine the factors which may influence the blastulation, and discuss their application in predicting of the pregnancy outcomes.

**Materials and Methods: **Day 3 (D3) after embryo transfer and freezing, surplus poor-quality embryos from IVF/ICSI cycles were cultured to blastocyst by the sequential method, then the blastulation outcomes were observed. Focusing on the blastulation rate of those embryos with different number cells and different embryonic grade; and last the relationship between the pregnancy outcomes of remained poor-quality embryos with successful blastulation or failed blastulation groups were studied.

**Results: **Of 127 patients with 569 poor-quality in vitro cultured embryos, there were formation of 248 blastocysts from 91 patients (43.59%), which lead to development of 138 high-quality blastocysts (24.25%). With the increase in cells number of D 3 blastomeres, the blastulation rate gradually increased, that, 7-cell blastomeres blastulation rate was the highest (70.59%), and 8-cell blastomeres is a little below (70.37%); while the embryonic levels and blastulation rate did not show this positive relationship. The clinical pregnancy rate and implantation rate of those who had successful blastulation (67.03% and 42.39%) were higher than of those who failed to develop to blastocyst (p=0.039).

**Conclusion:** Day 3 poor-quality embryos with successful blastulation or with failed blastulation had predictive value on pregnancy outcomes. For embryo transfer 7-8 cells grade III-IV embryo is better than 4-5 cells grade I-II embryo, in case of lack good-quality embryos.

## Introduction

With the development of human assisted reproductive technology, blastocyst transfer become more and more popular. In contrast with day 2-3 stage embryo transfers (Ets), the implantation rate is higher for blastocyst transfer (Day 2/3 22.6% vs. Day 5/6 32%) (1, 2). Single embryos transferred in selected patients, can be resulted in high pregnancy rates and reduced multiple gestations (3, 4). Because blastocyst development rate may vary however, some programs are reluctant to move to extended embryo culture in part due to fear that there may be no blastocyst to transfer on day 5 or 6 of culture, this may lead to psychological and economic problems. 

Currently, the majority of assisted reproductive centers use morphological score to assess the quality of the embryos, according to this, they choose the good-quality embryos for transfer into the uterine or cryopreservation after 2-3 days of in vitro fertilization (IVF). To optimize the benefit of those surplus embryos has become a problem (5). Those poor-quality embryos which have lower development potential and little significance of cryopreserved usually are discarded. Nowadays with the continuous development of in vitro culture techniques, the treatment of poor-quality embryos has been re-examined. Previous studies had reported that, those low morphological score embryos still have a potential to develop to blastocyst stage, and give rise to new (hESC) cell lines (6-13). This will solve the problem of the lack of sources of stem cell research. 

The embryos that develop to the blastocyst stage are influenced by many factors. Some studies demonstrated that: younger female age, increased parity, standard insemination, and lower doses of gonadotropins were significantly associated with good-quality blastulation formation (14). Another study compared between IVF and Intracytoplasmic sperm injection (ICSI) in blastulation rate that it estimated about 78% and 73% respectively (15). Moreover another study stated that, a co-culture of human embryos with endometrial epithelial resulting in blastulation rate of 50.8-58.2% (16). 

In 2010 Cetin *et al* reported that, the age is one of the important factors that affects IVF success, pregnancy rate of patients <35 years old was significantly higher than patients over 35 years old (17). Regarding implantation and pregnancy rate, uneven blastomere cleavage has a negative effect on both pregnancy and implantation rates (18). So far increasing of implantation rates of extended culture and blastocyst transfer are not limited to specific groups of patients or specific etiologies. Rather blastocyst transplant may be of benefit to the most if not all patients applying for IVF (19).

However few researches showed a comprehensive system studies on the poor-quality embryos cultured to blastocyst stage. So the objectives of the present study were: firstly, to observe the fate of those poor-quality embryos when they are cultured in vitro to blastocyst stage; secondly, to compare pregnancy rates based on the blastocyst development of remnant poor-quality embryos. We hope this information could then be useful in developing program protocols and in counseling patients before and during their cycle.

## Materials and methods

**Participants**

It was a retrospective and longitudinal study of couples who had undergone their first IVF/ICSI cycle at the First Hospital of Jilin University (half a year), inclusion and exclusion criteria: In the third day, patients were transfered two good quality embryos (defined in the following paragraph), the remaining good quality embryos were directly frozen; surpluses poor-quality embryos: if the embryos were 2PN at day one were continued culture to blastocyst; if the first day was non-2PN were discarded. 

Those couples, who had poor-quality embryos cultured to blastocysts stage were initially included in the study, excluding 26 couples, whose their wives were older than 35 years old, the final 127 couples of 569 poor-quality embryos met the inclusion criteria. Embryos which turn to blastocytes were first frozen, if the couples were not pregnant, we may use those blastocytes for transferred.

Good or poor quality embryos were evaluated according to the cell numbers of embryos and the embryos grade of day 3，only those 6-10 cells (day 2 must be 3-4 cells) and grade I-II embryos can be defined as the good quality embryos, and the rest were defined as poor-quality embryos. This study was approved by the Reproductive Medicine ethics committee of First Hospital of Jilin University, and all patients signed informed consents for poor-quality embryos blastocyst culture.

**Stimulus package and fertilization methods**

All patients were applied for IVF/ICSI and ET according to standard protocols. Cycles were generally initiated by a monthly oral contraceptive pills. Then all women exercised a luteal phase GnRH stimulation protocol with step-up gonadotropin dosing. HCG (5000 or 10,000 IU) was applied in case three or more follicles were >15 mm in diameter with the lead follicle R18 mm. Oocyte retrieval was performed 34-36 hours later. 

The laboratory techniques methodology were performed as described elsewhere (20). Fertilization was done 4-6 hours after retrieval with either intracytoplasmic sperm injection (ICSI) or conventional insemination as appropriate for the presence or absence of male factor.

**Embryos and blastocyst culture**

Fertilization and embryo culture were performed in Quinn's-1020 (SAGE, Inc. USA) medium enriched with 5% HAS (SAGE, Inc. USA) and Quinn's-1026 (SAGE, Inc. USA) medium with 10% serum protein substitute (SPS, SAGE, Inc. USA) respectively, the environment of the incubator is 37^o^C 5% CO_2_. At 17-19 hours after insemination, normal fertilization was confirmed by the presence of two pronuclei. Blastocyst culture medium used was 10% concentrated serum protein substitute (SPS) added to Quinn's-1029 (SAGE, Inc. USA) medium, which was incubated at 37^o^C with 6% CO_2_ and 89% N_2_.

**Standard of morphology score**

After 72 hours fertilization, according to modified PETER cleavage stage embryos scoring system to assess the day 3 embryo quality (21), the main indicators is the embryos size uniform or not, and how many fragments, the embryo is divided into four Grade, Grade I: uniform or slightly uneven with fragmentation of <10%; Grade II: the blastomeres size uniform or non-uniform, fragmentation amount of 10-20%; Grade III: an accounted embryo amount 21-50%; Grade IV: the amount of debris >50%. According to the criteria, the day 3 cell number 6-10 grade I and II embryos rated as high-quality embryos (D2 to D3 embryonic development of a blastomere except), the remaining is regard poor-quality embryos.

The blastocysts were classified for quality grades according to Gardner and Schoolcraft description (22, 23). This scoring system based on morphological evaluation of cell count, fragmentation, and embryo expansion, with the following grades (A, B, or C) describes both the trophectoderm and inner-cell mass of the embryo. Blastocyst quality was classified as good (≥4 BB), or poor according to its trophectoderm and inner-cell mass quality scores.

**Statistical analysis**

Statistical analysis was performed with SPSS version 17.0 statistical package (SPSS Inc., Chicago, IL, USA). Descriptive statistics were used to summarize all continuous and categorical variables. The association of patient and cycle characteristics with blastocyst development (results expressed as a percentage rate) was examined using χ^2^ test for Count data and Spearman Rank correlation analysis for Ranked data, α=0.05. p<0.05 indicates significant difference and all p-values were two-sided. 

## Results

The results showed that, out of 127 patients, there were 91 patients had successful blastulation formation (67.03%), with a total of 248 blastocysts formation (43.59%), including 138 good quality blastocysts (24.25%). The influence of female age less than 35 on blastulation rate was limited, and there was no significant difference between the age groups ≤25, 26-30 and 31-35, which estimated (46.02%, 40.71% and 44.91% respectively), (p-value see Table I). The blastulation rate between primary and secondary infertility also, was not statistically significant (44.25% and 42.13% respectively) (p=0.638), moreover in traditional IVF and ICSI groups also no significant different had been observed (46.19% and 41.91% respectively) (p=0.315) (Table I).

The results showed a closed positive relationship between the number of D3 blastomeres and blastulation, that with the increasing in the cells number of D3 embryos, blastulation rate shows an increasing trended from 2 cells embryos 0% (0/8) to 7-cells embryos 70.59%, 8-cells embryos 70.37% and 9 cells were 50.00%, and there was a positive correlation between the number of cells and the blastulation rate below 8-cells (p=0.000). 

**Figure 1:**
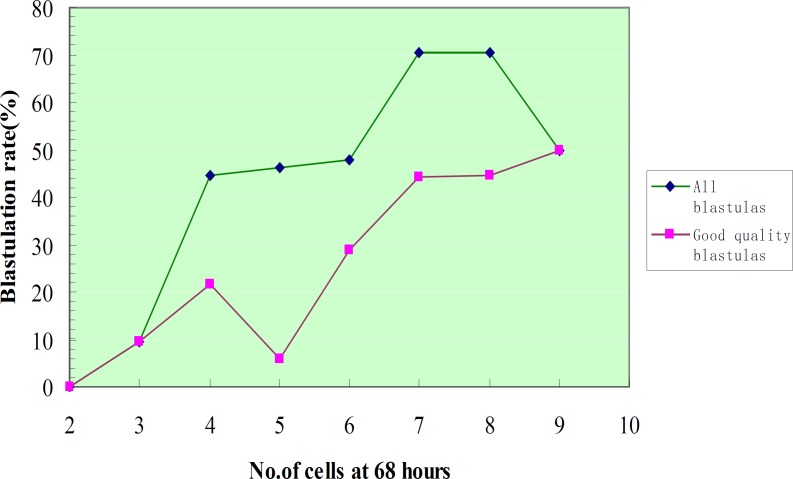
Blastulation rate according to the cell number of blastomeres at 68 hours after insemination

Further analysis of the relationship of the D3 III-IV embryonic blastomeres number and blastulation, the results showed that, increasing of the number of blastomeres followed by increasing in blastulation rate, 2-cell embryos had no successful blastulation; 7-cell was the highest, 48 successful blastulation out of 68 embryos (70.59%). The blastulation rate of odd number cells embryos compare with even number cells had no statistically significant difference (p=0.0530) (Figure 1, Table II).

Embryonic level of class III embryo has the highest blastulation rate of 56.95% (127/223), grade I and II embryonic blastulation rates were only 11.76% (4/34) and 37.06% (63/170) respectively. According to spearman correlation analysis, there was no correlation between the embryonic level and blastulation rate. For D3 4-5-cell embryos the study results showed a negative relation between the embryo quality and blastulation rate that; with the lower quality of embryo, the blastulation rate increased as 11.76% (4/34), 37.20% (61/164), 41.43% (29/70) and 49.32% (36/73) for grade I, II, III, and IV respectively (Table III). The pregnancy rate and implantation rate was about the good quality embryos which were transferred for the first time. The clinical pregnancy rate and implantation rates of those who had successful blastulation were 67.03% (61/91) and 42.39% (78/184) respectively, were higher than those who had no successful blastulation which pregnancy rate is 47.22% (17/36) and implantation rate was 29.49% (23/78). Which the difference was statistically significant in both groups (p=0.039，p=0.0497) (Table IV).

**Table I:** Blastulation rate of 569 poor-quality embryos for different groups

**Item**		**No. of embryos**	**Blastulation rate** *	**Good-quality blastocysts** *	**p-value**
Total number of embryos		569	248 (43.59%)	138 (24.25%)	
Female age (year)					
	≤25	176	81 (46.02%)	49 (27.84%)	0.2856 a
	26-30	226	92 (40.71%)	53 (23.45%)	0.8361 b
	31-35	167	75 (44.91%)	36 (21.56%)	0.4048 c
Etiology					
	Primary infertility	391	173 (44.25%)	92 (23.53%)	0.6378
	Secondary infertility	178	75 (42.13%)	46 (25.84%)	
Fertilization methods					
	In vitro fertilization	223	103 (46.19%)	58 (26.01%)	0.3148
	Intracytoplasmic sperm injection	346	145 (41.91%)	80 (23.12%)	

* Data are presented as n (%).

a : compare ≤25 and 26-30 group

b : compare ≤25 and 31-35 group

c : compare 26-30 and 31-35 group

**Table II:** Relationship between the cell number of day 3 poor-quality blastomeres and blastocyst formation

**Cell No. of blastomeres **	**No. of embryos**	**Blastulation rate** *	**Good-quality blastocysts** *	**Grade III-IV** ** embryos**		
				**No. of embryos**	**Blastulation rate** *	**Good-quality blastocysts** *
2	8	0 (0.00)	0 (0.00)	8	0 (0.00)	0 (0.00)
3	27	4 (14.81)	4 (14.81)	21	2 (9.52)	2 (9.52)
4	193	65 (33.68)	36 (18.65)	74	33 (44.59)	16 (21.62)
5	148	65 (43.92)	27 (18.37)	69	32 (46.38)	4 (5.80)
6	94	45 (47.87)	27 (28.72)	94	45 (47.87)	27 (28.72)
7	68	48 (70.59)	30 (44.12)	68	48 (70.59)	30 (44.12)
8	27	19 (70.37)	12 (44.44)	27	19 (70.37)	12 (44.44)
9	4	2 (50.00)	2 (50.00)	4	2 (50.00)	2 (50.00)
Even number	322	129 (40.06)	75 (23.29)	203	97 (47.78)	55 (27.09)
Odd number	247	119 (48.18)	63 (25.51)	162	84 (51.85)	38 (23.46)

There is a positive correlation between the cell number of blastomeres and the blastulation rate (p=0.000); Compare even number and odd number group, p= 0.0530.

* Data are presented as n (%).

**Table III:** Relationship between day 3 poor-quality embryonic grade and blastocyst formation

**Grade of embryos**	**No. of embryos**	**Blastulation rate** *	**Good-quality blastocysts** *	**Blastomere with 4-5 cells**		
				**No. of embryos**	**Blastulation rate** *	**Good-quality blastocysts** *
I	34	4 (11.76)	2 (5.88)	34	4 (11.76)	2 (5.88)
II	170	63 (37.06)	43 (25.29)	164	61 (37.20)	41 (25.00)
III	223	127 (56.95)	73 (32.74)	70	29 (41.43)	10 (14.29)
IV	142	54 (38.03)	20 (14.08)	73	36 (49.32)	10 (13.70)

* Data are presented as n (%).

**Table IV:** Influence of successful blastulation on pregnancy and implantation rate

**Group**	**Cycles**	**Pregnancy rate** *	**Implantation rate** *
Formation blastocyst	91	61 (67.03)	78.184 (42.39)
Non-formation blastocyst	36	17 (47.22)	23.78 (29.49)
Total	127	78 (61.42)	101.262 (38.55)
p-value		0.0387	0.0497

* Data are presented as n (%).

## Discussion

In this study 248 out of 569 poor-quality embryos had successful blastulation (43.59%), including 138 good quality blastocyst (24.25%). The blastulation rate, of primary or secondary infertility, traditional IVF, ICSI, and different age group (<35) had no significant difference (p-value see Table I). The D3 cell count is an important indicator of embryo assessment, a lot of studies have reported a relationship between the speed of embryonic development and blastulation, most of these studies demonstrated a positive relationship between blastomeres number and blastulation rate. This relation is limited, that, when embryos had more than 8 cells, blastocyst development did not further increase (24-26). 

A previous study by Alikani *et al* had reported that, D3 (day 3) 7-9 cells cleavage, the embryonic blastulation rate was 41.9%, while in case of less than 7 and more than 9 cells, embryonic blastulation rate is decreased significantly (27). In 2003 Neuber* et al* found a significant positive relationship between early cleaving 2-cell embryos and subsequent good quality ≥4-cell, ≥7-cell and blastocyst development (28). Our study is agreed with the previous studies which had revealed a positive relationship between the number of blastomeres and blastulation rate, with the highest rate of 7-cell embryos (70.59%), followed by 8-cell embryos (70.37%). 

We further analyzed the D3 III-IV embryos with different blastomeres number of blastulation circumstances, the result is similar to above finding, that whatever quality of embryo, 7-cell embryos blastulation rate is the highest at 70.59% (48/68). The odd number embryos shows a better blastulation rate than even number embryos (48.18% vs. 40.06%), but has no statistically significant differences (p=0.053), for the unrevealed reason, future studies are needed. The D3 embryos with greater cells number are not always predictive of a high blastulation rate, that when the D3 embryo had more than 8 cells the blastocyst development did not longer increase (24, 25). This fact is in accordance with our study, but because the embryos with cell count >9 is very few (only 4 embryos) in our study, we cannot deduce to that conclusion. 

In our study, the D3 high quality embryos may not have the highest rate of blastulation, but embryonic level of class III embryo has the highest blastulation rate of 56.95% (127/223), grade I and II embryonic blastulation rate was only 11.76% and 37.06% respectively. By further study in 4-5-cell blastomeres subgroup, we found that, blastulation rate had a negative correlation with embryo quality. Analyzing the possible causes of above result, grade I and II embryos less than 6-cells, likely undergo diapause in the second day (in our study 76% of the embryos were growth less than one cell in D2-D3), those undeveloped embryos may had a very low development potential. Scientists had observed that mouse eggs often stop to develop at the 2-cell stage, this phenomenon is known as the 2-cell block (cell block), 2-cell block mechanism is still unknown (29). 

Of course, many factors affect the formation of the blastocyst, here is also worth more depth to explore. For grade III and IV embryos may have a self-healing capability, and eventually get a good rate of blastulation. Our study also showed that 7-8cells grade III-IV embryos blastocyst formation rate is significant high than 4-5cells grade I-II embryos, with blastulation rate 70.59%, 70.37% vs. 11.76%, 37.20% respectively. So if there was no good quality embryos to transfer, 7-8-cells grade III-IV embryos is better than 4-5 cells grade I-II for transfer. The number of blastomeres is an important factor which affects subsequent embryo developmental potential. When we transfer or further culture the selected type of embryos with 7-8 cells, there would be a greater chance to obtain a better outcome. It has been proposed that the blastulation of surplus embryos in IVF treatment can directly predict pregnancy outcome (26, 30-31).

In 2000, Racowsky *et al* when they compared the outcomes of day 3 and day 5 embryo transfer concluded that, if there has no 8-cell embryo on day 3, transfer at day 3 is warranted. With one or two 8-cell embryos, any benefit of day 5 transfer seemed to be equivocal. With three or more 8-cell embryos day 5 transfer is recommended (32). Our result also showed a predict value of pregnancy rate on remained poor-quality embryos which had successful blastulation or fail to develop to blastula. The clinical pregnancy rate (CPR) of those who had successful blastulation (67.03%) is significant higher than those who failed to develop to blastocysts (47.22%) (p=0.0387). The implantation rate (IR) of those who had successful blastulation (42.39%) is significant higher than those who had failed to develop to blastocysts (25.00%) (p=0.0497). 

It means that, those couples who had successful blastulation, their embryos may have a better development potential. But the pregnancy rate and implantation rate are also influenced by many other factors like receptivity of the endometrium, luteal support, hormone levels, etc., (not only the embryo’s developmental potential). A large number of studies have shown that discarded embryos can be used as the source of the hESC line establishment (6-13). The experimental results for how to select the discarded embryos develop to provide a basis for: 1) The number of blastomeres may be more, but better below 8; 2) The younger female age; 3) Embryo grading III and IV with high cell number may also be a good choice. 

This can effectively alleviate the establishment of the problem of lack of embryos in the process of human embryonic stem cell bank and reduce ethics condemn. The inadequacies of this study are that the small number of samples used in the present study, and although we have done our best to exclude influence factors, but we cannot exclude all those factors. In the future, we will increase the study sample, refinement study provides more powerful clinical data guide, and improve ethical oversight, and the development of the application of the remaining embryos, such as the creation of embryonic stem cell bank.

In summary, our research made a systematic analysis of the poor-quality embryos blastocyst culture, we found a positive relation between the number of cells in the day3 embryo and blastulation rate. But the embryos grade had no relationship with the blastulation rate, may be due to embryonic diapause. Day3 poor-quality embryos with successful blastulation or with failed blastulation had predictive value on clinical pregnancy rate and implantation rate. 7-8 cells grade III-IV embryos are better than 4-5 cells grade I-II embryos for embryo transfer. In case there is no good-quality embryos available for transfer, 7-8 cells grade III-IV embryos is recommended. Those who had blastulation of poor-quality embryos may have a better clinical pregnancy rate and implantation rate.

## References

[1] Blake DA, Farquhar CM, Johnson N, Proctor M (2007). Cleavage stage versus blastocyst stage embryo transfer in assisted conception. Cochrane Database Syst Rev.

[2] Papanikolaou EG, Kolibianakis EM, Tournaye H, Venetis CA, Fatemi H, Tarlatzis B (2008). Live birth rates after transfer of equal number of blastocysts or cleavage-stage embryos in IVF. Hum Reprod.

[3] Gardner DK, Surrey E, Minjarez D, Leitz A, Stevens J, Schoolcraft WB (2004). Single blastocyst transfer: a prospective randomized trial. Fertil Steril.

[4] Ryan GL, Sparks AET, Sipe CS, Syrop CH, Dokras A, Van Voorhis BJ (2007). A mandatory single blastocyst transfer policy with educational campaign in a United States IVF program reduces multiple gestation rates without sacrificing pregnancy rates. Fertil Steril.

[5] Hoffman DI, Zellman GL, Fair CC, Mayer JF, Zeitz JG, Gibbons WE (2003). Cryopreserved embryos in the United States and their availability for research. Fertil Steril.

[6] Mitalipova M, Calhoun J, Shin S, Wininger D, Schulz T, Noggle S (2003). Human embryonic stem cell lines derived from discarded embryos. Stem Cells.

[7] Suss-Toby E, Gerecht-Nir S, Amit M, Manor D, Itskovitz-Eldor J (2004). Derivation of a diploid human embryonic stem cell line from a mononuclear zygote. Hum Reprod.

[8] Chen H, Qian K, Hu J, Liu D, Lu W, Yang Y (2005). The derivation of two additional human embryonic stem cell lines from day 3 embryos with low morphological scores. Hum Reprod.

[9] Lerou PH, Yabuuchi A, Huo H, Miller JD, Boyer LF, Schlaeger TM (2008). Derivation and maintenance of human embryonic stem cells from poor-quality in vitro fertilization embryos. Nat Protoc.

[10] Feki A, Bosman A, Dubuisson JB, Irion O, Dahoun S, Pelte MF (2008). Derivation of the first Swiss human embryonic stem cell line from a single blastomere of an arrested four-cell-stage embryo. Swiss Med Wkly.

[11] Geens M, Mateizel I, Sermon K, Rycke MD, Spits C, Cauffman G (2009). Human embryonic stem cell lines derived from single blastomeres of two 4-cell stage embryos. Hum Reprod.

[12] Cortes JL, Sanchez L, Ligero G, Gutierrez-Aranda I, Catalina P, Elosua C (2009). Mesenchymal stem cells facilitate the derivation of human embryonic stem cells from cryopreserved poor-quality embryos. Hum Reprod.

[13] Ström S, Rodriguez-Wallberg K, Holm F, Bergström R, Eklund L, Strömberg A (2010). No relationship between embryo morphology and successful derivation of human embryonic stem cell lines. PLos One.

[14] Thomas MR, Sparks AE, Ryan GL, Van Voorhis BJ (2010). Clinical predictors of human blastocyst formation and pregnancy after extended embryo culture and transfer. Fertil Steril.

[15] Westphal LM, Hinchley MD, Behr B, Milki AA (2003). Effect of ICSI on subsequent blastocyst development and pregnancy rates. J Assist Reprod Genet.

[16] Mercader A, Garcia-Velasco JA, Escudero E, Remohí J, Pellicer A, Simón C (2003). Clinical experience and perinatal outcome of blastocyst transfer after coculture of human embryos with human endometrial cells: a 5-year follow-up study. Fertil Steril.

[17] Cetin MT, Kumtepe Y, Kiran H, Seydaoglu G (2010). Factors affecting pregnancy in IVF: age and duration of embryo transfer. Reprod Biomed Online.

[18] Hardarson T, Hanson C, Sjögren A, Lundin K (2001). Human embryos with unevenly sized blastomeres have lower pregnancy and implantation rates: indications for aneuploidy and multinucleation. Hum Reprod.

[19] Schoolcraft WB, Gardner DK (2001). Blastocyst versus day 2 or 3 transfer. Semin Reprod Med.

[20] Dokras A, Baredziak L, Blaine J, Syrop C, Van Voorhis BJ, Sparks A (2006). Obstetric outcomes after in vitro fertilization in obese and morbidly obese women. Obstet Gynecol.

[21] Brinsden PR (1999). A textbook of in vitro fertilization and assisted reproduction.

[22] Gardner DK, Schoolcraft WB, Jansen R, Mortimer D (1999). In-vitro culture of human blastocysts. Towards reproductive certainty: fertility and genetics beyond 1999.

[23] Gardner DK, Lane M, Stevens J, Schlenker T, Schoolcraft WB (2000). Blastocyst score affects implantation and pregnancy outcome: towards a single blastocyst transfer. Fertil Steril.

[24] Shapiro BS, Harris DC, Richter KS (2000). Predictive value of 72-hour blastomere cell number on blastocyst development and success of subsequent transfer based on the degree of blastocyst development. Fertil Steril.

[25] Langley MT, Marek DM, Gardner DK, Doody KM, Doody KJ (2001). Extended embryo culture in human assisted reproduction treatments. Hum Reprod.

[26] Luna M, Copperman AB, Duke M, Ezcurra D, Sandler B, Barritt J (2008). Human blastocyst morphological quality is significantly improved in embryos classified as fast on day 3 (≥10 cells), bringing into question current embryological dogma. Fertil Steril.

[27] Alikani M, Calderon G, Tomkin G, Garrisi J, Kokot M, Cohen J (2000). Cleavage anomalies in early human embryos and survival after prolonged culture in-vitro. Hum Reprod.

[28] Neuber E, Rinaudo P, Trimarchi JR, Sakkas D (2003). Sequential assessment of individually cultured human embryos as an indicator of subsequent good quality blastocyst development. Hum Reprod.

[29] Hardy K, Stark J, Winston RM (2003). Maintenance of the inner cell mass in human blastocysts from fragmented embryos. Biol Reprod.

[30] Balaban B, Urman B, Sertac A, Alatas C, Aksoy S, Nuhoglu A (1998). Progression of excess embryos to the blastocyst stage predicts pregnancy and implantation rates after intracytoplasmic sperm injection. Hum Reprod.

[31] Fisch JD, Milki AA, Behr B (1999). Sibling embryo blastocyst development correlates with the in vitro fertilization day 3 embryo transfer pregnancy rate in patients under age 40. Fertil Steril.

[32] Racowsky C, Jackson KV, Cekleniak NA, Fox JH, Hornstein MD, Ginsburg ES (2000). The number of eight-cell embryos is a key determinant for selecting day 3 or day 5 transfer. Fertil Steril.

